# The benefits of looking at intraindividual dynamics in cognitive training data

**DOI:** 10.3389/fpsyg.2015.00615

**Published:** 2015-05-12

**Authors:** Tanja Könen, Julia Karbach

**Affiliations:** ^1^Department of Psychology, Goethe UniversityFrankfurt, Germany; ^2^Center for Research on Individual Development and Adaptive Education of Children at Risk (IDeA)Frankfurt, Germany

**Keywords:** working memory, training, intraindividual variability, micro-longitudinal, within-person, trajectories, fluctuations, learning disabilities

Over the last decade, the prospect of improving or maintaining cognitive functioning has provoked a steadily increasing number of cognitive training studies. Central target populations are individuals at risk for a disadvantageous development, such as older adults exhibiting cognitive decline or children with learning impairments. They rely on cognitive resources to meet the challenges of an independent life in old age or requirements at school.

To support daily cognitive functioning, training outcomes need to generalize to other cognitive abilities. Such transfer effects are, however, highly discussed. For example, recent meta-analyses on working memory training differed in the conclusion on the presence (Au et al., 2015; Karbach and Verhaeghen, 2014) or absence of transfer effects (Melby-Lervåg and Hulme, 2013). Usually training-specific design factors such as type, intensity, duration, and feedback routines are discussed as reasons for such inconsistent findings. However, even individuals participating in exactly the same training regime highly differ in their training outcomes. We argue that it is time to study the individual development during trainings to understand these differential outcomes. It is time to have a closer look at the intraindividual training data.

## Within-person information in training data

The classical findings of a training study – whether a cognitive training group showed training and transfer effects compared to a control group – could be amended and sometimes even better understood by further analyzing the training sessions on the within-person level. Intraindividual training data could offer four types of information: (1) *Intraindividual performance trajectories* across all training sessions can demonstrate which participants show training effects and when they reach their performance maximum. (2) *Intraindividual performance fluctuations* – between and within training sessions – show which participants vary substantially in their performance (despite general training improvement). (3) *Intraindividual couplings* of performance fluctuations with other variables can reveal which internal and external factors contribute to individual performance and to what extend participants differ in the strength of these relations. (4) *Further combinations* of these types can be considered as well. For example, substantial performance fluctuations (type 2) can in theory be both, an indicator of adaptive (e.g., varying strategies; Siegler, 1994, 2007) or maladaptive processes (e.g., vulnerability to disturbing influences) during training. Relating fluctuations to other variables such as daily motivation and affect (type 3) or to performance trajectories (type 4, here combining 1 and 2) can contribute to exposing them as either beneficial or obstructive for the individual training success.

## Rationale of the approach

There is a strong rationale behind this approach of looking at the intraindividual and dynamic characteristics of cognitive training data. Until recently, the training phase of intervention studies constituted a black box and we gradually tested hypothesized mechanisms through the variation of training conditions. Combining this tradition with within-person analyses is a more efficient approach to understanding how cognitive training works, for whom it works, and in which contexts and situations it works by combining the benefits of two different research perspectives. The cognitive training field provides cumulating evidence for the role of individual differences in training (e.g., Lövdén et al., 2012; Karbach and Unger, 2014, for a review), where some individuals benefit more than others. Most of the time, however, one can only speculate about the underlying mechanisms that lead to these differences. Measurement intensive research, such as the field of ambulatory assessment, provides cumulating evidence that individuals differ in intraindividual cognitive processes. They differ in the strength of cognitive performance fluctuations (i.e., short-term variations) and their couplings with possible antecedents and consequences (e.g., Riediger et al., 2014; Könen et al., 2015).

Combining both perspectives is reasonable, because a central assumption behind cognitive training is that it fosters intraindividual change in cognitive performance. But how exactly do individuals come from A to B (i.e., pre to post level)? Dynamic systems theory predicts that a later state of cognition (y_t+1_) is a function of an earlier state (y_t_) with the function being an adaptive mechanism to perturbation (y_t+1_ = *f*(y_t_); cf. Weisstein, 1999; van Geert and Steenbeek, 2005). In our case, the perturbation would be a challenging cognitive training and individuals do respond to this situation. Ideally, they develop new cognitive resources because they experience a prolonged mismatch between their resources and the situational demands (cf. Lövdén et al., 2010). Practically, some individuals gain more than others, even if the training is adaptive and well designed (thus, neither too easy, nor too difficult). Individuals likely vary in within-person processes over time that eventually produce between-person differences in training outcomes. Consequently, it is just a logical step to look at the intraindividual level to find out what happens over the course of the training.

## Implications for specific populations

A within-person approach to cognitive training data is all the more beneficial the more heterogeneous the trained individuals are. Good examples to illustrate this point are specific populations, such as children with learning disabilities and older adults. On the one hand, they demonstrate between group differences compared to healthy controls or young adults, and on the other hand they likely exhibit substantial within group differences. Such differences are of major concern because they can influence training outcomes or even mask effects. Therefore, both populations are particularly useful to highlight the benefits of a within-person approach.

Learning disabilities constitute an important target for cognitive training, because they have been related to substantial working memory impairments (e.g., Schuchardt et al., 2008; Fischbach et al., 2014). However, the profile of these impairments varies considerably between disabilities (e.g., reading vs. spelling disability, Brandenburg et al., 2014). Varying impairments can influence training outcomes because the initial performance level is often related to training and transfer gains (e.g., Zinke et al., 2013; Karbach et al., 2014). Consequently, individuals sharing a specific learning disability might not only function differently from healthy controls, but also from those with other learning disabilities. Further, they likely show a substantial amount of within-group variability and may, for example, vary in the etiology of the learning disability and with regard to possible treatments they previously received (cf. Shah et al., 2012). In cases with such crucial heterogeneity, within-person analyses can reveal *whether and to what extent* participants perform differently in the course of a cognitive training (i.e., show differential intraindividual effects). For example, they might show different cognitive performance trajectories and different antecedents and consequences of performance fluctuations (i.e., differ in the internal and external factors contributing to individual performance). A related within-person finding comes from a sample of elementary school children. Daily working memory performance was related to last night's sleep quality and this within-person coupling varied reliably between children. It was stronger for low performing children, indicating that they were more vulnerable to the influence of last night's sleep (Könen et al., 2015).

Further promising examples for the usefulness of within-person analyses come from training research with older adults. Older adults demonstrated on average a slower growth during working memory training than younger adults (Bürki et al., 2014) and their performance fluctuated less in all tasks of a broad cognitive training (working memory, episodic memory, and processing speed, Schmiedek et al., 2013). In addition, their performance fluctuations in reasoning and perceptual speed were positively associated with practice-related gains on the same tasks (Allaire and Marsiske, 2005), implying that fluctuations likely indicated an adaptive process in this case. Taken together, these examples demonstrate that the performance of older adults during cognitive trainings differs from the performance of younger adults, which is valuable additional information on top of between-person differences in training outcomes (e.g., in Schmiedek et al., 2010). They suggest a need to question what causes these differences and whether older adults' behavior can be modified through, for example, instruction and feedback (e.g., Garrett et al., 2012). Interestingly, the within-person relation between daily motivation and daily working memory training performance was considerably lower in older compared to younger adults (Brose et al., 2010), raising the question whether there are untapped motivational resources and whether the effectiveness of cognitive trainings for older adults may be improved by building more on these motivational resources. Still, much more research is needed to further confirm and elaborate these first recent findings.

## Statistical modeling

Suitable modeling approaches are, for example, multilevel modeling (e.g., Brose et al., 2012; Schmiedek et al., 2013), structural equation modeling (SEM; e.g., Brose et al., 2010; Bürki et al., 2014), dynamical systems analysis (e.g., Gasimova et al., 2014), and combinations thereof (e.g., multilevel SEM, e.g., Könen et al., 2015). We suggest using or at least starting with multilevel models because they are easy to access and to apply (e.g., closely related to standard regression, implemented in all commonly used software packages) and they are perfectly suitable to study intraindividual trajectories, fluctuations, and couplings. In these models, a certain number of measurement occasions (e.g., training sessions, Level 1, within-person level) are nested within a certain number of individuals (Level 2, between-person level). One can, for instance, predict a Level 1 variable (e.g., daily cognitive performance) with another Level 1 variable (e.g., daily motivation) and test the mean intraindividual effect (fixed effect) and examine whether individuals (Level 2) differ reliably in the strength of this relation (random effect). Hoffman and Stawski (2009) provide a detailed discussion of multilevel analyses with longitudinal data.

## Practical considerations

There is already a number of existing cognitive training studies with data suitable for all or at least a part of the proposed within-person analyses (e.g., Bürki et al., 2014). We want to encourage the field to further explore the potential of the existing data and to consider within-person processes when designing future training studies. Therefore, one should pay particular attention to the number of measurement occasions, the sample size, and the sensitivity of the measures to fluctuations.

The number of measurement occasions (*K*) and the sample size (*N*) should be reviewed together in a multilevel context. Cognitive trainings are expected to change cognitive performance on a construct level, so the frequency of training sessions (here: *K*) is usually high. Whether a given *K* is sufficient for a certain within-person analysis depends on *N* as well as the size and nature of the effect of interest (e.g., an intraindividual coupling). The typical 10–20 training sessions applied in cognitive trainings (cf. Melby-Lervåg and Hulme, 2013, Appendix) are sufficient for within-person analyses if *N* is appropriate. The first step to calculate the necessary *N* would be to conduct a traditional power analysis concerning the central transfer effects of the training (e.g., with G^*^Power; Faul et al., 2007). Then one could use the resulting *N* of trained participants as a lower starting point for Monte Carlo simulations on the multilevel parameters (to estimate the probability of recovering known population parameters given *K* and *N*; for example with Mplus; see Bolger et al., 2012; Bolger and Laurenceau, 2013; for a step-by-step description). One should simulate different combinations of *K* and *N* and could also consider other design factors (e.g., the number of observed indicators) to find an optimal trade off and study design (cf. von Oertzen and Brandmaier, 2013). In case an existing *N* is slightly lower than preferred, Bayesian estimation could be eligible (Hox et al., 2012).

Variables that might impact daily cognitive performance (e.g., current motivation, affect, and health) should be observed with every training session, if feasible. This allows for the estimation of couplings over time. For instance, Brose et al. (2012) found that working memory performance during a cognitive training in young adults was lower on days with reduced motivation, reduced control of attention, and enhanced negative affect. To allow for such analyses, one has to consider the temporal dynamics of the variables and carefully select measures that are sensitive to fluctuations. For example, an affect scale has to capture the current state and should not include items for rare affective states. The variables of interest could be assessed with short scales (see Ziegler et al., 2014) to reduce testing time and participant burden. The reliability of these scales and their sensitivity to fluctuations can be analyzed with multilevel models (e.g., Wilhelm and Schoebi, 2007). We highly recommend the handbook of Mehl and Conner (2012) for a detailed and elaborate introduction to measurement intensive research.

## Summary and outlook

Cognitive training data could offer more information than is currently used in the field. We suggest analyzing intraindividual performance trajectories, fluctuations, and couplings and to consider such within-person analyses when designing future training studies. This seems to be particularly promising for studies with heterogeneous samples. Individuals likely vary in within-person effects over time that eventually produce between-person differences in training outcomes. Thereby, a within-person approach could contribute to understanding training outcomes and to generating theories about the underlying mechanisms.

Some hypotheses could then be further tested and validated through classic variations of training conditions. Experimental variation is the only way to ensure valid causal inferences in cognitive psychology. However, it is practically impossible to test all thinkable explanations for the current heterogeneous findings in training research only in this way. It seems much more feasible to test certain mechanisms that were already identified in the intraindividual dynamics of the training data. This highlights how both perspectives complement each other and could be combined to an efficient approach to study the mechanisms that drive or hamper cognitive training success.

### Conflict of interest statement

The authors declare that the research was conducted in the absence of any commercial or financial relationships that could be construed as a potential conflict of interest.
